# A Bird’s-Eye View of the Pathophysiologic Role of the Human Urobiota in Health and Disease: Can We Modulate It?

**DOI:** 10.3390/pathophysiology31010005

**Published:** 2024-02-01

**Authors:** Emilio Jirillo, Raffaele Palmirotta, Marica Colella, Luigi Santacroce

**Affiliations:** 1 Interdisciplinary Department of Medicine, Section of Microbiology and Virology, School of Medicine, University of Bari “Aldo Moro”, 70124 Bari, Italy; emilio.jirillo@uniba.it (E.J.); raffaele.palmirotta@uniba.it (R.P.); luigi.santacroce@uniba.it (L.S.); 2 Doctoral School, eCampus University, 22060 Novedrate, Italy

**Keywords:** microbiota, urobiota, gut, immunity, urinary tract infections, antibiotics

## Abstract

For a long time, urine has been considered sterile in physiological conditions, thanks to the particular structure of the urinary tract and the production of uromodulin or Tamm–Horsfall protein (THP) by it. More recently, thanks to the development and use of new technologies, i.e., next-generation sequencing and expanded urine culture, the identification of a microbial community in the urine, the so-called urobiota, became possible. Major phyla detected in the urine are represented by *Firmicutes*, *Bacteroidetes*, *Proteobacteria*, and *Actinobacteria.* Particularly, the female urobiota is largely represented by *Lactobacillus* spp., which are very active against urinary pathogenic *Escherichia (E.) coli* (UPEC) strains via the generation of lactic acid and hydrogen peroxide. Gut dysbiosis accounts for recurrent urinary tract infections (UTIs), so-called gut–bladder axis syndrome with the formation of intracellular bacterial communities in the course of acute cystitis. However, other chronic urinary tract infections are caused by bacterial strains of intestinal derivation. Monomicrobial and polymicrobial infections account for the outcome of acute and chronic UTIs, even including prostatitis and chronic pelvic pain. *E. coli* isolates have been shown to be more invasive and resistant to antibiotics. Probiotics, fecal microbial transplantation, phage therapy, antimicrobial peptides, and immune-mediated therapies, even including vaccines for the treatment of UTIs, will be described.

## 1. Introduction

Current investigations into human microbiota and microbiome have greatly contributed to better understanding the beneficial roles of microorganisms, which colonize many districts of our body [1]. Alteration of the equilibrium between protective and pathogenic bacteria (dysbiosis), e.g., in the gut and in the urinary tract, may lead to a condition of disease. The link between microbiota and metabolic/immune activities represents the major focus of this emerging field [2,3,4,5]. In particular, the bacterial components of the microbiota can, via their metabolic products, directly or indirectly modulate the function of many cell types, even including immune cells, ultimately eradicating pathogens.

The old belief that urine is a sterile environment has prevented for a long time further investigation of the presence of the urinary microbiota, the so-called urobiota. Only over the last decade has the existence of a complex microbial community in the urine been acknowledged [6,7]. In this regard, the use of new technologies, such as next-generation sequencing and expanded quantitative urine culture, has allowed for new accomplishments to be made in the field of urobiota [8]. According to Shoemaker, in urine samples, the most common bacteria detected are the following: *Lactobacillus*, *Streptococcus*, *Gardnerella*, *Staphylococcus*, and *Corynebacterium.* In addition, other bacteria, such as *Burkholderia*, *Klebsiella*, *Prevotella*, and *Veilonella*, are detectable, even at lower levels [9], thus contributing to the microbial diversity that is the major feature of human urine, whose composition depends on several factors. For instance, in pre-menopausal women, *Lactobacillus* spp. are very dominant, while in post-menopausal women, *Mobiluncus* levels are increased [10].

Also, aging seems to exert its impact on the composition of urobiota, since in individuals over 70 years of age, uncommon bacteria are found in the urine, i.e., *Proteiniphilum*, *Saccharofermentans*, and *Parvimonas* [11,12,13,14].

Other factors, which may contribute to differences in the composition of the urobiota, are represented by the collecting mode of urinary samples. From the analysis of a series of reports, it appears that suprapubic aspiration and transurethral catheterization are the best modalities for collecting samples since both procedures avoid contamination from the genitals [15,16,17,18].

From a pathogenic point of view, shifts in the microbial community of the urobiota may lead to a urinary tract infection (UTI) [19,20]. In this respect, the prevalence of uropathogenic strains of *Escherichia coli* may account for chronic infections. Women are affected by UTIs more than men, due to a shorter urethra and hormonal changes, as in the case of lower estrogen amounts in post-menopausal women [21,22,23].

Furthermore, the abuse of antibiotic treatment often causes alterations in the urobiota with the development of antimicrobial resistance and chronic recurrent cystitis [24,25].

Quite interestingly, the gut microbiota has been shown to account for the majority of UTIs, with *Escherichia (E.) coli* as the prevalent uropathogen [26]. Through clonal tracking, evidence has been provided that UTIs are very often preceded by an exaggerated growth of uropathogens [27]. Moreover, a link between vaginal and urinary microbial strains (*Lactobacillus*, *E. coli*, and *Streptcoccus (S.) anginosus*) has been reported, thus indicating the coexistence of a urogenital microbiota in women [28].

Recurrent (r) UTIs are mainly caused by uropathogenic *E. coli* (UPEC) strains, which colonize the periurethral area, then move up to the urethra, and ultimately reach the bladder [14,29,30,31]. Recurrent UTIs are very often monomicrobial infections, but in some cases, they are polymicrobial infections with *Enterococcus (E.) faecalis* and UPEC strains acting as major etiologic factors [32]. Polymicrobial biofilms characterize rUTIs with *E. faecalis*, *Proteus (P.) mirabilis*, and *Klebsiella (K.) pneumoniae* as dominant organisms [33,34]. Furthermore, polymicrobial interactions in the context of biofilms are responsible for antibiotic resistance, i.e., against ciprofloxacin and trimethoprim [35,36].

Chronic prostatitis (CP) is very common in men under age 50, and some studies have reported an increase in *Burkholderia cenocepacia* in the course of CP [37,38,39]. Another study by Shoskes reported the association of CP with the gut microbiota also in view of higher counts of *Clostridia* and *Bacteroidia* in CP patients in comparison to controls [40].

Antibiotic therapy still represents the best treatment to combat UTIs. However, the misuse or abuse of antibiotics has permitted the occurrence of multi-antibiotic-resistant bacteria [41].

Therefore, different approaches are currently under investigation in order to restore the subverted urobiota [42].

*E. coli* 83972 has been shown to compete with UPEC isolates, and its introduction in neurogenic bladders decreased the frequency of UTIs. Moreover, *Lactobacillus (L.) cispatus* administration was very effective in the prevention of UTIs [43,44].

Also, fecal microbiota transplantation in patients with rUTIs decreased the colonization of multi-antibiotic-resistant bacterial strains, while increasing antibiotic susceptibility to uropathogens [45].

Among bacterial inhibitors, experimental evidence has been provided that mannosides operate as receptor analogs and prevent the binding of UPEC fimbriae to epithelial cells in the bladder [46]. Another strategy is based on the administration of ceragenins that are able to enhance the function of endogenous LL-37, an antimicrobial peptide (AMP) [47,48].

The aim of the present review is to describe the urobiota functions in health and disease, also to provide information on novel therapeutic approaches to combat UTIs, with probiotics and bacterial by-products, especially in the case of antibiotic resistance. Finally, immune-mediated therapeutic attempts will be described, even including the application of new vaccines.

## 2. The Composition of the Urobiota

The urobiota is composed of a core of bacteria with four major phyla, *Firmicutes*, *Bacteroidetes*, *Proteobacteria*, and *Actinobacteria* [49]. Of note, *Actinobacteria* and *Bacteroidetes* are lacking in the male urobiota, with *Corynebacterium* as a dominant strain [50].

In general terms, microbial composition male urobiota resembles that present in the skin microbiota [51]. Moreover, changes in the male urobiota composition also depend on concurrent disease, since *Propionibacterium acnes* strains are prevalent in patients with prostatic cancer, whereas *Enterobacteriaceae* and *Actinobacteria* dominate in benign prostate tumors [52,53].

The female urobiota resembles that of the vaginal microbiota with a prevalence of *Lactobacillus* spp. [54]. *Lactobacillus* spp. play a protective role against UPEC pathogens via their by-products, such as lactic acid and hydrogen peroxide [55,56]. Conversely, in pre-menopause women, reduced levels of *Lactobacillus* correlate with urinary incontinence as an indication of urobiota imbalance [57,58]. Of note, female urobiota resembles that of the skin during menstruation, while after sexual intercourse, an increase in *Staphylococcus* and *Streptococcus* has been reported [59].

**Figure 1:**
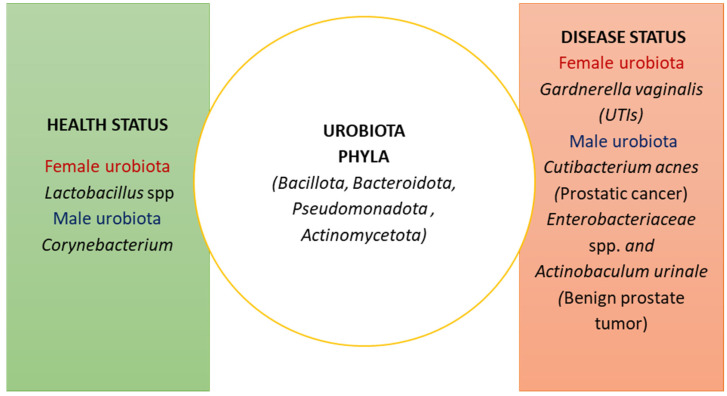
Composition of the urobiota in health and disease. In female urobiota, *Lactobacillus* spp. play a protective role against urinary pathogenic *Escherichia coli* (UPEC) strains via release of lactic acid and hydrogen peroxide, while *Gardnerella vaginalis* may cause a re-emergence of UPEC strains in experimental UTIs. In male urobiota, *Propionibacterium acnes* and *Enterobacteriaceae* and *Actinobaculum urinale* are associated with malignant and benign prostate tumor, respectively. Taken together, these data indicate the different roles of urobiota phyla, ranging from protection to induction of infections and/or tumors.

*Gardnerella (G.) vaginalis*, that is very abundant in bacterial vaginosis, correlates with a decrease in *Lactobacillus* spp. when it is present at high levels in the female urobiota, thus exposing women to UTI development, pyelonephritis, and renal infections [60,61]. The composition of the urobiota is depicted in Figure 1. 

## 3. The Impact of the Gut Microbiota on UTI Occurrence

Several pieces of evidence support the concept that the gut microbiota is involved in the pathogenesis of UTIs [62,63,64]. For instance, increased levels of *Enterobecteriaceae* and *E. coli* in the gut have been associated with UTI recurrence [65,66].

UPEC strains can colonize the urinary tract in view of their surface factors and secreted virulence factors. Among surface factors, type 1 and P fimbriae account for adhesion to host cells, biofilm formation, and the release of cytokines, respectively [67]. On the other hand, secreted virulence factors comprise hemolysins, cytotoxic necrotizing factors 1, and siderophores, which are responsible for intracellular survival, iron acquisition, and tissue damage [68,69].

Extraintestinal pathogenic *E. coli* (ExPEC), when ingested with food and/or water, are neutralized by the gut epithelium, but in the case of intestinal epithelium dysfunction, they overcome the gut barrier and translocate to the urinary tract [70].

Moreover, antibiotic-induced dysbiosis facilitates the translocation of ExPEC to extraintestinal sites, even including the urinary tract [71]. Quite interestingly, endotoxemia resulting from gut dysbiosis has been detected in the course of stress and depression associated with rUTIs [72]. Clinically, women with uncomplicated rUTIs have been found to have gut dysbiosis with decreased levels of *Lactobacillus* spp. and *Bifidobacteria* along with increased numbers of *E. coli*, *Clostridium*, and *Staphylococcus* spp. [72]. In support of the above data, a gut–bladder syndrome has been reported, thus indicating the passage of pathogens from the gut to the bladder [73]. 

Recently, a positive association has been reported between urobiota and overactive bladder, a neuromuscular dysfunction predominated by the presence of urinary urgency, suggesting a possible role of urinary dysbiosis in this common condition. However, it seems to be worsened by an overlapping viral infection that can also affect bacterial diversity and composition. There are very limited studies with non-uniform results, but a constant feature is the difference in the microbial population of the gut and genito-urinary tract between individuals with urge urinary incontinence and healthy people. For further information, readers should refer to the recent studies conducted by [74,75,76].

## 4. Urobiota Alteration in UTIs and Pathogenetic Mechanisms

In the course of UTIs, a variety of bacteria have been identified in urobiota by current DNA sequencing methods. According to Price et al. and Kenneally et al., in aged people, immunocompromised individuals, patients with indwelling devices, and those who have received previous antibiotic treatment, urobiota is characterized by a dramatic decrease in *E. coli*, *Enterococcus*, *Staphylococcus*, *G. vaginalis*, and *Lactobacillus*, paralleled by an increase in *Pseudomonas aeruginosa*, *Aerococcus urinae*, and *Proteus mirabilis* [74,75]. Within this framework, there is evidence that bacteria originating from the gastrointestinal tract or urogenital epithelial niches create the so-called intracellular bacterial communities (IBCs) in the bladder wall, as observed in acute cystitis and rUTI patients [76]. 

UPEC strains contributing to the formation of IBCs belong to phylogroups A, B1, B2, C, E, F, and D, with B2 and D endowed with stronger virulence and higher levels of multidrug resistance [77,78].

Thanks to modern technologies, it has been discovered that UTIs also constitute polymicrobial infections with *E. faecalis* and UPEC strains as dominant bacteria [79].

Of note, L-ornithine is secreted by *E. faecalis* with the subsequent stimulation of *E. coli’s* iron uptake pathway and related biofilm formation [79,80]. Also, catheter-associated UTIs (CAUTIs) are polymicrobial in nature, and *E. coli* isolates are more invasive with increased resistance to antibiotics [81]. In the same direction, *P. mirabilis* and *E. faecalis* biofilms are resistant to various antibiotics, such as aminoglycosides, fluroquinolones, and carbapenems [82]. In particular, *P. mirabilis* produces urease, leading to crystalline biofilm formation on catheters and resistance against pre-coated antimicrobial agents [83]. In sum, the above cited pathogenetic mechanisms account for disease progression and more severe adverse effects in comparison to monomicrobial infections [84].

Prostate infections are caused by various bacterial strains, i.e., *E. coli*, *Pseudomonas*, *Klebsiella*, *Enterococcus*, *Enterobacter*, *Proteus*, and *Serratia*, and can be divided into acute bacterial, chronic bacterial, asymptomatic inflammatory prostatitis, and chronic pelvic pain [85].

The above pathogens replace the prostate microbiota, that is mainly represented by *Proteobacteria*, *Firmicutes*, *Actinobacteria*, and *Bacteroidetes* [86,87,88]. Despite the fact that the prostate is not connected to the gut microbiota, it can be reached by intestinal post-biotics, such as endotoxins or lipopolysaccharides (LPSs), from the cell wall of Gram-negative bacteria [89].

In this respect, prostate cancer cells express on their membrane Toll-like receptor (TLR)4, that is the specific receptor for LPSs and whose activation accounts for the release of proinflammatory cytokines, vascular endothelial growth factor, and CCL2 [90,91]. Furthermore, *E. coli* strains isolated from chronic prostatitis patients modify the prostatic milieu, promoting tumor growth [92]. Conversely, evidence has been provided that small RNAs isolated from *Pseudomonas* spp. are not correlated with prostate cancer development and may retard metastatic dissemination [93].

From a pathogenic point of view, immune response in the course of prostatitis is able to promote a massive liberation of proinflammatory cytokines and enzymes, e.g., collagenase or glucuronidase, that contribute to prostate damage [94]. 

The relationship between urobiota and bladder cancer has been the subject of a previous investigation in terms of bacteria which may promote tumor growth. However, in a recent review, the role of urinary bacteria able to arrest cancer progression has been pointed out with special reference to their capacity to reinforce immunosurveillance [95]. The authors conclude that the exact mechanisms of the anti-neoplastic protection afforded by bacteria require further study. 

**Figure 2:**
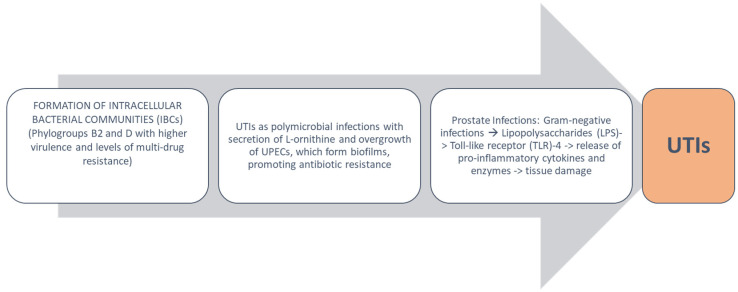
Pathogenesis of UTIs. IBCS are endowed with more virulence and higher levels of resistance to antibiotics. In polymicrobial infections, secretion of L-ornithine causes overgrowth of UPECs and resistance to antibiotics, as in the case of catheter-associated UTIs. In prostate infections, secretion of LPSs maintains the chronicity of disease.

The pathogenesis of UTIs is expressed in Figure 2.

## 5. New Therapeutic Approaches to Modulate the Urobiota and Prevent UTIs

Until recently, antibiotic treatment has represented the most appropriate therapy to defeat UTIs. However, the misuse and abuse of antibiotics have increased the number of antibiotic-resistant uropathogens, thus affecting the microbicidal activity of these drugs. For this reason, novel therapeutic approaches are under investigation to restore the urobiota [96,97]. 

### 5.1. Probiotics

Probiotics have largely been used for correcting the gut microbiota in view of their ability to inhibit pathogen colonization, modulate host immune response, and maintain epithelial barrier integrity [98,99,100]. Therefore, probiotics have also been used in UTIs. For instance, *Lactobacillus* spp., when administered via intravaginal suppository, could prevent the outcome of UTIs [101]. *L. crispatus* is able to secrete lactic acid, which is highly microbicidal against *C. trachomatis (C.t.)*, as well as *Candida albicans* [102,103,104].

*L. rhamnosus* has been shown to be very effective in UTIs, downregulating UPEC virulence, even including NF-kB activation, P, and type 1 fimbriae, as well as preventing the formation of biofilms [105,106,107]. Moreover, the intravesical administration of *L. rhamnosus* has been demonstrated to be beneficial in patients with neurogenic lower urinary tract infections [108].

*E. coli* Nissle 1917, a non-pathogenic strain, is currently used as a preventative probiotic agent against UTIs in view of its ability to antagonize other uropathogens [109].

Actually, this bacterium possesses catechol microcins, which support bacterial competitiveness by binding ferric iron under iron-limited circumstances [110,111].

*E. coli* 83972 is an attenuated strain, which differentiates from the *E. coli* strains detected in UTIs, thanks to *fim* deletions and *papG* point mutations, which reduce its motility and account for the absence of P, type 1, and F1c fimbriae [112]. This bacterium is able to colonize the urinary tract, outperforming uropathogens and decreasing UTI risk [113,114]. 

Another avirulent *E. coli* strain, HU217, has been used to coat urinary catheters, thus reducing the incidence rates of infection in patients [115].

Of note, both prebiotics and synbiotics have been used in a few clinical trials in patients with UTIs [116,117]. Further studies are needed to validate the efficacy of these agents.

### 5.2. Fecal Microbiota Transplantation

Fecal microbiota transplantation (FMT) is based on the transfer of healthy stool to the intestinal lumen of a given patient with the attempt to restore the antibiotic-mediated subversion of the gut microbiota. Application of the FMT in patients with recurrent *Clostridioides difficile* infection and UTIs has led to an increased antibiotic susceptibility of the uropathogens, *E. coli* and *Klebsiella*, so it has been proposed for selected patients [118]. Similarly, in patients with irritable bowel syndrome and rUTIs, FMT has reduced pathogenic strains in stool, while it has also attenuated UTI symptoms [119].

### 5.3. Phage Therapy

Phage therapy for UTIs is based on the use of lytic proteins, phage cocktails, and phages associated with antibiotics [120]. In a human murine model, the combination of a phage with antibiotics (TMP-SMX and ciprofloxacin) reduced the biofilm biomass and a multi-drug-resistant strain of *Acinetobacter baumannii* [121]. The synergistic effect seems to rely on the inhibition of dihydrofolate reductase, DNA topoisomerase II and IV, with a faster phage expansion and bacterial cell lysis [122]. Also, the association between anti-*Pseudomonas* phages and *E. coli* HU2117, a derivative of *E. coli* 83972, was very effective in the prevention of *P. aeruginosa* biofilm in urinary catheters [123].

Pyophage, a commercial preparation, has been shown to be very effective in the treatment of UTIs [124,125]. The intravesical administration of Pyophage in patients with UTIs has been very effective as an antibiotic treatment, even if further trials are required to fully validate this type of treatment [125].

### 5.4. Antimicrobial Peptides

AMPs are produced by the urothelium in the urinary tract, and, among them, human cathelicidin (LL-37), human-beta defensin (HBD), and human-alpha defensin 5 (HD-5) have been clinically evaluated. Selected probiotics and nutraceuticals could be used to induce an increased production and release of these molecules, offering new therapeutic and prophylactic tools for urinary diseases [126,127,128,129,130,131].

LL-37 exerts protective effects in the course of UTIs, and, in particular, it suppresses biofilm formation by the inhibition of *CsgA* polymerization, a subunit of curly fimbriae, which generates resistance in UPC strains [132,133,134].

Alpha-defensins possess an elevated antimicrobial potential, and the recombinant HD5 has been shown to be highly microbicidal against several uropathogens [135].

HBD-1 and HBD-2 are present in the urinary tract, and, in particular, HBD-2 is very effective against *E. faecalis* [136]. Cecropin A, derived from the wax moth, *Galleria Mellonella*, has been demonstrated to be active against biofilm-forming UPEC strains [137]. However, more research is needed to define the potential of HBDs in the urinary tract. 

### 5.5. Bacteriocins

Bacteriocins also belong to AMPs and are defined as ribosomal synthetized peptides by both Gram-positive and Gram-negative bacteria [138]. The dramatic increase in multi-drug-resistant Gram-negative bacteria has prompted more research in the field of Gram-negative bacteriocins. Colicins and microcins are major Gram-negative bacteriocins, with the former having a higher molecular weight (>20 kDa) and the latter being characterized by a lower molecular weight (<10 kDa).

Colicins are synthetized by more than 50% *E. coli* strains and employ various mechanisms to destroy bacteria, i.e., membrane permeabilization via voltage-dependent channels, cellular nuclease degradation, and the inhibition of peptidoglycan synthesis [139]. Clinically, colicins are able to reduce biofilm generation in urinary catheters, and their antibacterial activity can be increased, combining a lubricant with colicin SR4 or using *L. brevis* DT24, which expresses colicin E2 [140,141,142]. Furthermore, bioengineered nisin variants S29A and S29G, model bacteriocins, exhibited a higher potency against *Shigella* and *Pseudomonas* spp. [143,144,145].

Pheromonicin results from the combination of a channel-forming colicin 1a and its cognate immunity protein from *E. coli* with a pheromone-encoding gene from *S. aureus* and exhibits specific activity against *S. aureus* [146].

Chimeric bacteriocins have also been demonstrated to be very effective in the killing of *E. coli* and *P. aeruginosa* [147,148].

Microcins are AMPs encoded by plasmids or chromosomes and show antibacterial activity against *Enterobacteriaceae* spp. [149]. They act via the inhibition of bacterial enzymes, membrane permeabilization, and the exploitation of membrane components [149,150]. The insertion of microcin H47 and microcin M into *E. coli* Nissle 1917 enhanced their activity against UPEC strains [151,152].

In terms of bioengineering, the insertion of non-canonical amino acids into microcin J25 gave rise to several variants with similar or even higher microbicidal activity in comparison with the wild counterpart [153,154]. Also, the combination of chitosan nanoparticles with microcin J25 generated a stable nano-antimicrobial effective against both Gram-positive and Gram-negative bacteria [155].

**Figure 3:**
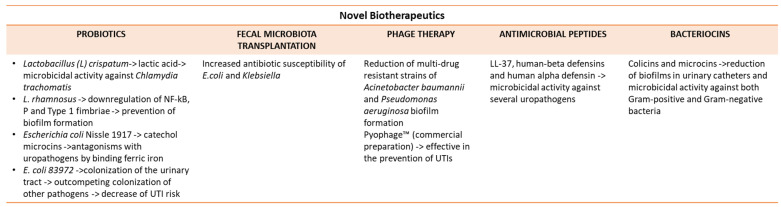
Bacteria and bacterial by-products as novel biotherapeutics. Probiotics and fecal microbiota transplantation are very active in preventing the risk of UTIs, outperforming uropathogens. Phages are active in combination with antibiotics or *E. coli* Hu2117 in the prevention of UTIs. Antimicrobial peptides and bacteriocins are bacterial by-products with microbicidal potential against uropathogens.

In Figure 3, bacteria and bacterial by-products as novel biotherapeutics are described.

### 5.6. Inhibitors of Bacterial Virulence

Mannosides are small molecules able to function as receptor analogs that can bind fimbriae, thus preventing UPEC-host cell interactions, as seen in murine infection models and human kidney cells [156,157,158]. Then, targeting bacterial type 1 pilus fimh adhesins with mannosides selectively depleted uropathogenic *E. coli* from the gut, potentially reducing UTI incidence [159].

### 5.7. Immune-Mediated Therapies

There is evidence that LL-37 accounts for neutrophil, macrophage, and T lymphocyte responsiveness in UTIs [160]. In this respect, synthetic antimicrobials, ceragenins, have been shown to enhance LL-37 activity in the urinary tract [161,162]. Moreover, a prolyl-hydroxylase inhibitor, GB-004, was able to regulate the hypoxia-inducible factor-1 alpha, reducing the inflammatory infiltrate in murine UTIs [163]. The above cited studies suggest the possibility of reinforcing the immune response or decreasing the inflammatory profile in the urinary tract. 

IL-22, a member of the IL-10 cytokine superfamily, has been shown to regulate the microbiota and the expression of AMPs in human urothelial cells [164,165]. According to the studies of Sonnenberg et al. and Schirinzi et al., IL-22 may be used to control UTI infections, reducing the inflammatory burden [165,166].

Vaccines composed of UPEC antigens have been proposed, such as ExPEC4V, which targets the O-polysaccharide chain of a pathogenic strain of *E. coli* LPSs in women rUTIs [167]. Furthermore, the sublingual spray vaccine, Uromune, composed of heat-inactivated lysates of *E. coli*, *E. faecalis*, *K. pneumonia*, and *P. vulgaris*, could prevent UTIs in women, with only 10% of the vaccinated group undergoing UTIs within 12 months after vaccination [168].

**Figure 4:**
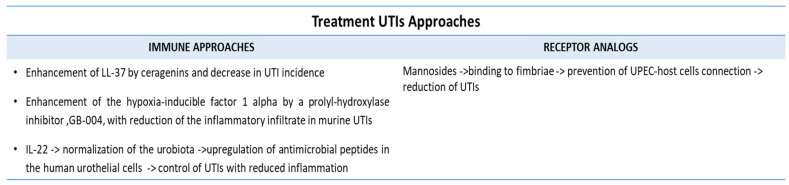
Use of receptor antagonists, immune molecules, and vaccines in the prevention of UTIs. Mannosides prevent the binding of fimbriae to host cells, thus reducing the incidence of UTIs. LL-37, hypoxia-inducible factors, and IL-22 are immune molecules that are able to reduce the inflammatory infiltrate in the course of UTIs. ExPEC4V and Uromune vaccines are under investigation for the prevention of UTIs in women.

In Figure 4, molecular and immune-mediated approaches to treat UTIs are described.

## 6. Conclusions

The urobiota represents the microbial community of the urinary tract. It interacts with the microbiota of the gastrointestinal and genital tracts, regulating local and systemic immune responsiveness. Dysbiosis of the urobiota can lead to UTIs, which may be complicated by antibiotic-resistant bacterial strains. In this regard, several therapeutic attempts are at present being investigated for the restoration of the urobiota. Besides biotherapeutics, i.e., probiotics and FMT procedure, immune-mediated approaches are under study, using AMPs and targeting cytokines, such as IL-22, for controlling inflammation, as well as bacterial colonization during UTIs. Finally, an emerging field is represented by the use of vaccines that target the O-polysaccharide chain of ExPEC LPSs with encouraging results in rUTI women, even if further clinical data are necessary to validate vaccine efficacy.

## Data Availability

All relevant data are reported in the manuscript.

## List of Abbreviations

CAUTI	Catheter-associated UTI
CP	Chronic prostatitis
ExPEC	Extraintestinal pathogenic *Escherichia coli*
FMT	Fecal Microbiota Transplantation
HBD	Human-beta defensin
HD5	Human-alpha defensin 5
IBCs	Intracellular bacterial communities
IL	Interleukin
LPSs	Lipopolysaccharides
R	Recurrent
UTIs	Urinary tract infections
UPEC	Uropathogenic *Escherichia coli*
Th	T helper
TREG	T regulatory cells
